# Thermodynamics of the Glassy Polymer State: Equilibrium and Non-Equilibrium Aspects

**DOI:** 10.3390/polym16020298

**Published:** 2024-01-22

**Authors:** Costas Panayiotou

**Affiliations:** Department of Chemical Engineering, Aristotle University of Thessaloniki, 54624 Thessaloniki, Greece; cpanayio@auth.gr

**Keywords:** statistical thermodynamics, lattice-fluid, glass densification, glass transition, polymer swelling, penetrant sorption

## Abstract

This work examines, first, the non-equilibrium character of the glassy state of polymer systems and its significance in the development of novel materials for important technological applications. Subsequently, it summarizes the essentials of the generalized lattice fluid approach for the description of this highly complex non-equilibrium behavior with an approximate and simple, yet analytically powerful formalism. The working equations are derived in a straightforward and consistent manner by clearly defining the universal and specific variables needed to describe the discussed properties. The role of the non-random distribution of molecular species and free volume in the glassy system is also examined, as is the role of strong specific interactions, such as hydrogen-bonding networks. This work also reports examples of applications in a variety of representative systems, including glass densification, retrograde vitrification, increase in glass-transition temperature in hydrogen-bonded polymer mixtures, and hysteresis phenomena in sorption–desorption from glassy polymer matrices.

## 1. Introduction

High polymers are encountered in the crystalline, amorphous or semi-crystalline state. The amorphous state may be further differentiated into a series of states, from a viscous liquid state at high temperatures, to a rubbery state at intermediate temperatures, to a glassy state at lower temperatures. There are excellent monographs and reviews on the broader aspects of the glassy polymer state in the open-source literature [1,2,3,4]. The focus in this work will be on the aspects of the glassy polymer state and its peculiarities with regard to thermodynamics. Both the glassy state and the (rubber-to-) glass transition of high polymers will be discussed. For this purpose, some key characteristics differentiating the liquid state from the glassy polymer state should be recalled first.

In the melt state above the glass transition temperature, Tg, high polymer chains are typically entangled and exhibit a broad spectrum of relaxation times. These relaxation times can be attributed to a variety of modes of motion, i.e., bead-spring Rouse dynamics and reptation in entangled systems [5], as well as smaller-scale vibrational and rotational motions. On cooling from the melt state and approaching Tg, the material reaches a rubbery viscoelastic state, with molecular conformations kept in equilibrium via local bead-spring Rouse modes of motion between entanglement points and other local segmental relaxation modes. The glass transition represents to a high degree the dynamic arrest of these local, cooperative segmental modes of motion. In the glassy state below Tg, the polymer chains are effectively locked, and only small-scale rotational and vibrational modes of motion are active. The consequence of this change is well known: the modulus of elasticity of the glassy polymer increases by three orders of magnitude.

**Figure 1:**
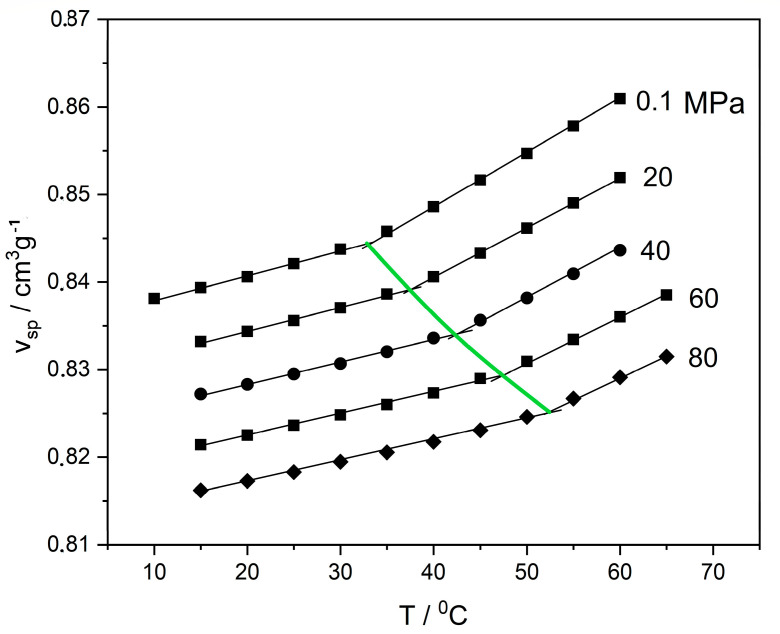
Experimental PVT data for poly(vinyl acetate) (PVAc) at the glass transition region [6]. The straight lines are drawn to show the change in slope at the glass transition and the definition of Tg at the intersection. The thick green line passing through the intersections shows the variation in Tg with pressure.

**Figure 2:**
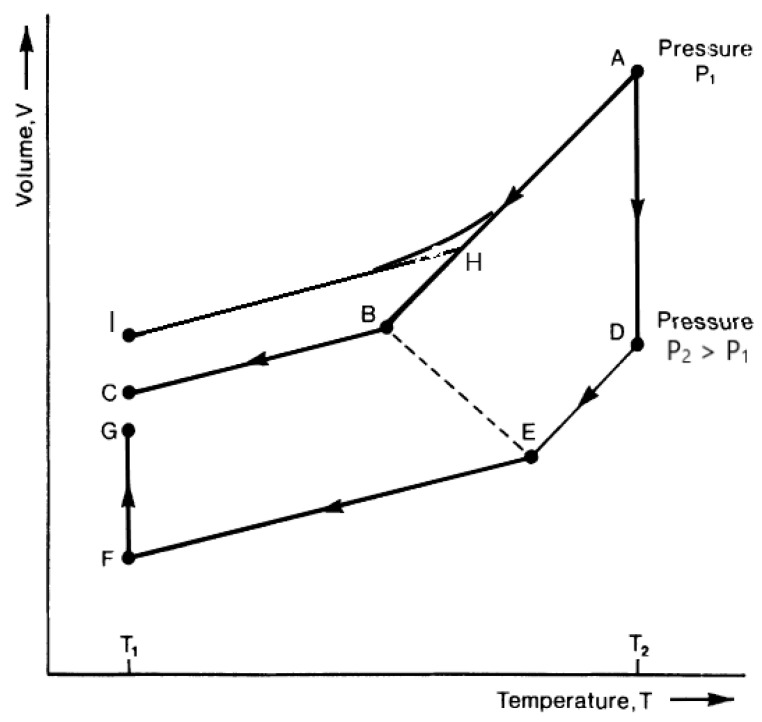
Schematic illustration of the role of formation history on the density of the final glassy state. States I and C are obtained from the same isobars (same constant pressure, atmospheric) at different cooling rates. State G, shown at the same final temperature *T*_1_, is obtained by first pressurizing the melt to a high pressure, P_2_, and then cooling isobarically to temperature *T*_1_, at which point isothermal depressurization takes place until atmospheric pressure is reached.

Regarding thermodynamic behavior, the transition from the viscous/rubbery state to the glassy state is a transition from non-equilibrium to equilibrium molecular conformations, implying a transition from the domain of equilibrium thermodynamics to the domain of non-equilibrium thermodynamics. This transition is associated with a series of peculiar phenomena [1,2,3,4,5]. Some of these peculiarities, relevant to the present work, are shown in Figure 1 and Figure 2. Figure 1 shows typical experimental pressure–volume–temperature (PVT) data in the glass-transition region of poly(vinyl acetate) (PVAc) [6]. The reported data were obtained by slow cooling rate at 5 °C/h. A distinct slope change is shown at the glass-transition temperature, Tg, at each isobar. As shown, the pressure has a significant effect on Tg, with increased Tg at higher pressures. This increase is simultaneously accompanied by a volume reduction or density increase in the final glassy state (glass densification).

However, the density of the final glassy state depends on the history of its formation, as shown in Figure 2. Faster cooling leads to a higher Tg and a lower final glass density. In contrast, pressurization and isobaric cooling followed by isothermal pressure release results in a denser material in the final glassy state [2]. Thus, as shown in Figure 2, the very same polymer at the very same temperature, T_1_, and pressure (atmospheric) exhibits three different densities at the three final glassy states: I, C, and G. The higher density of state G could be obtained, in principle, by very slow cooling at atmospheric pressure, but that process could take hundreds of years, compared to a few hours for the indicated pressurization–cooling–depressurization process [7].

The direct consequence of the points above is that the glassy state is not an equilibrium state, so the Gibbs phase rule of equilibrium thermodynamics does not apply. Different combinations and ranges of volumes, enthalpies, free energies, etc. may be compatible with a given *T*–*P* pair for a glassy polymer. Even if the density, *ρ*, of a particular polymer glass at a specific *T*–*P* pair for specific external conditions has been measured precisely, this *ρ*(*T*, *P*) value may change with time due to relaxations in the internal structural. Similarly, the glass transition is not a classical thermodynamic phase transition. Gee [1] conducted a systematic study on the thermodynamic character of the glass transition in oligomers and polymers. If the glass transition were an equilibrium phenomenon, the following equalities would apply (1st and 4th Ehrenfest equations):

$$\frac{dT_{g}}{dP}=\frac{\Delta \kappa_{T}}{\Delta \alpha }=\frac{TV\Delta \alpha }{\Delta C_{p}}$$

where Δ*α*, Δ*κ_T_*, and Δ*C_p_* are the changes that occur in the transition from the liquid to the glassy state of the thermal expansion coefficient, the isothermal compressibility and the heat capacity, respectively. Rather than fitting the above Equation, carefully conducted measurements on polystyrene samples gave the following results [1]:

$$\frac{dT_{g}}{dP}=3100\left[\frac{K}{Nm^{-2}}\right],\;\;\frac{\Delta \kappa_{T}}{\Delta \alpha }=7100\left[\frac{K}{Nm^{-2}}\right],\;\;\frac{TV\Delta \alpha }{\Delta C_{p}}=3600\left[\frac{K}{Nm^{-2}}\right]$$

The large discrepancy between the second ratio and the other two is a further indication that the polymer glass transition is not an equilibrium thermodynamic phase transition. The glass transition exhibits discontinuous changes in quantities that are second-order derivatives of Gibbs free-energy, *G*, such as the heat capacity and the thermal expansion coefficient. As the extent of these changes is not what would be expected for an equilibrium transition, the glass transition is often referred to as a *quasi-second-order* phase transition*,* with both thermodynamic and kinetic character.

As mentioned above, in terms of mechanical strength, the glass transition is associated with a drop of three orders of magnitude in the elasticity modulus with the change from the relatively strong glassy polymer state to the elastic rubber state. Thus, *T_g_* is important for practical applications of the polymer, as well as for its modification into advanced materials, such as microcellular foams or microporous scaffolds for tissue-engineering applications [8,9,10,11]. In foaming applications, supercritical fluids at moderately high pressures are extensively used as foaming agents. These fluids act also as plasticizers of the polymer matrix, thus reducing its *Tg*. As seen above, however, the application of high pressure causes an increase in *Tg*. The subtle interplay of these two competing effects leads to a variety of interesting 2-D glass transition profiles, including the technologically interesting phenomenon of retrograde vitrification [12,13]. Compressed fluids are used in numerous polymer processes, such as polymer impregnation, extraction, or fractionation [14]; membrane conditioning [15]; spray painting [16]; etc.

Apart from the interaction of compressed fluids with glassy polymers, another challenging issue for thermodynamic analysis is the involvement of strong specific interactions in the polymer-penetrant system [3,17,18,19], as well as in the glass transition behavior of polymer mixtures [18,20]. In terms of statistical thermodynamics, these interactions significantly influence the radial distribution function and the non-random distribution of the interacting segments in the system. Naturally, the non-random distribution of the free volume in the glassy polymer system is also associated with these interactions.

The problems described above are not trivial even for the rubbery or liquid polymer state. It is clear, however, that an equation-of-state approach would be a more appropriate thermodynamic approach for addressing these issues coherently. Indeed, one of the most successful approaches for handling glassy-polymer/fluid systems is the NET-GP (non-equilibrium thermodynamics for glassy polymers) model created by Doghieri and Sarti [3,21,22], which operates within the framework of the lattice fluid (LF) equation-of-state theory [23,24,25,26].

Thus, the central objective of this work is to review the equation-of-state approach to the glassy polymer state. For coherence of presentation, this work will focus primarily on the LF approach. The next section provides first a summary and general overview of the essentials of the LF model, then discusses its application to the glass transition. Subsequently, the thermodynamic basis and rationale of the NET-GP approach are critically reviewed and discussed.

## 2. The LF Equation-of-State Model and the Glass Transition

In the first part of this section, the basics of the LF model will be presented. Details may be found in the relevant literature [23,24,25,26]. In the second part of this section, the LF model will be applied to the glass transition, with emphasis on the glass transition of polymer–compressed-fluid systems.

### 2.1. The LF Model

In LF model, each of the *N_k_* molecules of type *k* in the system is characterized by a number *r_k_* of segments of hard-core volume, v_k_*, and of ε_k_* average interaction energy per segment. These segments are considered to be arranged on a quasi-lattice of *N_r_* sites, *N_0_* of which are empty. For clarity of presentation, we will confine ourselves to the case of binary mixtures. The extension of this formalism to multicomponent mixtures is straightforward [18,23,24,25,26]. For a binary mixture, we may write the following balance equation:

*N_r_ = r*_1_*N*_1_ + *r*_2_*N*_2_ + *N*_0_ = *rN* + *N*_0_ (1)

If v* is the volume of each lattice site, the total volume is given by

*V = (rN + N*_0_)v* = V* + N_0_v* (2)

Thus, V* is the occupied volume, while *N*_0_v* is the unoccupied or free volume. Segment fractions in LF are defined by

(3) $$\phi_{1}=\frac{x_{1}r_{1}}{r}=1-\phi_{2}$$

When the segment volumes of pure components do not differ significantly, the segment volume, v*, of the mixture is given by

(4) $$v^{*}=\phi_{1}v_{1}^{*}+\phi_{2}v_{2}^{*}$$

When $v_{1}^{*}$ differs significantly from $v_{2}^{*}$, alternative mixing and combining rules are used [4,5,6,7]. 

For practical applications, the LF model provides two relevant key equations: the equation of state and the equation for the chemical potential. The equation of state is used for the estimation of the system volume or density at the external temperature and pressure and for the given composition. The equation for the chemical potential or fugacity is used for the estimation of solubility or sorption of the fluid in the glassy polymer under the specified external conditions. Both of the above equations are derived from the LF equation for the Gibbs free energy, which is as follows:

(5) $$\frac{G}{RT}=rN\left\{-\frac{\tilde{\rho }}{\tilde{T}}+\frac{\tilde{P}\tilde{v}}{\tilde{T}}+\left(\tilde{v}-1\right)\ln\left(1-\tilde{\rho }\right)+\frac{1}{r}\ln\tilde{\rho }+\sum_{k=1}^{2}\frac{\phi_{k}}{r_{k}}\ln\frac{\phi_{k}}{\omega_{k}}\right\}$$

$\tilde{P},\tilde{T}$ and $\tilde{\rho }$ are the reduced pressure, temperature and density, respectively, which are defined as follows:

(6) $$\tilde{P}=\frac{P}{P^{*}}=\frac{Pv^{*}}{\varepsilon^{*}},\;\;\;\tilde{T}=\frac{T}{T^{*}}=\frac{RT}{\varepsilon^{*}},\;\;\tilde{\rho }=\frac{\rho }{\rho^{*}}$$

with the average segmental interaction energy, ε*, given by

(7) $$\varepsilon^{*}=\phi_{1}\varepsilon_{1}^{*}+\phi_{2}\varepsilon_{2}^{*}-\phi_{1}\phi_{2}RTX_{12}$$

*X*_12_ is given by

(8) $$X_{12}=\frac{\varepsilon_{1}^{*}+\varepsilon_{2}^{*}-2\varepsilon_{12}^{*}}{RT}$$

with

(9) $$\varepsilon_{12}^{*}=\xi_{12}\sqrt{\varepsilon_{1}^{*}\varepsilon_{2}^{*}}$$

The parameters ε*, v*, and r or, alternatively, the parameters T*, P*, and ρ*, are the three *scaling constants* or equation-of-state parameters of each liquid [18,23,24,25,26]. ξ_12_ is a binary adjustable parameter close to unity. The reduced volume $\tilde{v}$ is often used instead of the reduced density and is defined by

(10) $$\tilde{v}=\frac{V}{V^{*}}=\frac{N_{r}}{rN}=\frac{\rho^{*}}{\rho }=\frac{1}{\tilde{\rho }}$$

The LF equation of state is obtained from the free-energy-minimization condition with respect to the reduced density or to the number of empty sites in the system, at equilibrium. This equation remains identical in form for all cases of pure components and mixtures and is as follows:

(11) $$EOS=\frac{\tilde{P}\tilde{v}}{\tilde{T}}+\left[\frac{\ln\left(1-\tilde{\rho }\right)}{\tilde{\rho }}+\frac{\tilde{\rho }}{\tilde{T}}+1\right]-\frac{1}{r}=0\left(\mathrm{EOS}\;=\;0\;\mathrm{at}\;\mathrm{equilibrium}\right)$$

The chemical potential of component 1 in the mixture is, similarly, obtained from the derivation of Gibbs free energy with respect to N_1_, and is given by

(12) $$\begin{array}{l} \frac{\mu_{1}}{RT}=\ln\phi_{1}+\left(1-\frac{1}{r}\right)\phi_{2}+r_{1}\tilde{\rho }X_{12}\phi_{2}^{2}+r_{1}\left(\tilde{v}-1\right)\ln\left(1-\tilde{\rho }\right)+\ln\frac{\tilde{\rho }}{\omega_{1}}-\frac{r_{1}\tilde{\rho }}{{\tilde{T}}_{1}}+r_{1}\frac{\tilde{P}\tilde{v}}{\tilde{T}}\frac{v_{1}^{*}}{v^{*}}\\ -rN\tilde{v}\frac{\partial \tilde{\rho }}{\partial N_{1}}EOS \end{array}$$

It should be stressed that Equation (11) holds true at equilibrium. Thus, at equilibrium, the last term on the right-hand side of Equation (12) is zero and may be neglected. This assertion does not hold true, however, at non-equilibrium conditions, as for glassy polymer systems. In these latter cases, this term is non-zero and should be retained.

The corresponding equation for the chemical potential of pure compound **1** (fluid/penetrant), is:

(13) $$\frac{\mu_{1}^{0}}{RT}=r_{1}\left({\tilde{v}}_{1}-1\right)\ln\left(1-{\tilde{\rho }}_{1}\right)+\ln\frac{{\tilde{\rho }}_{1}}{\omega_{1}}-\frac{r_{1}{\tilde{\rho }}_{1}}{{\tilde{T}}_{1}}+r_{1}\frac{{\tilde{P}}_{1}{\tilde{v}}_{1}}{{\tilde{T}}_{1}}$$

At equilibrium, the chemical potential of the penetrant (component **1**) in the gas phase (Equation (13)) is equal to its chemical potential in the polymer phase (Equation (12)).

### 2.2. The Glass Transition

For This study of glassy polymers, Equation (12) must be considered with a focus on the term containing the chain-flexibility/symmetry parameter, *ω_i_*. This parameter is given by the following equation [18,23,24,25,26,27,28]:

(14) $$\omega_{i}=\delta_{i}/2e^{r_{i}-1}$$

The flexibility term, *δ_i_,* is a characteristic temperature-dependent property of each polymer and is given by [13,29]

(15) $$\delta_{i}=z{\left(\frac{z-2}{f_{i}}\right)}^{\left(r_{i}-2\right)f_{i}}{\left(\frac{1}{1-f_{i}}\right)}^{\left(r_{i}-2\right)\left(1-f_{i}\right)}$$

The term $2e^{r_{i}-1}$ that is the denominator to *δ_i_* in Equation (14) is a correction factor arising from the excluded volume of the chain in the system. The ‘*flex factor*’, *f_i_*, is used to indicate the *f_i_(r_i_−*2) bonds of the chain, which are in high-energy or flexed states, and the (1*−f_i_*)(*r_i_−*2) bonds, which are in the low-energy states. If Δ*ε_g_*_i_ is the energy change upon bond flexing (say, from a ‘trans’ to a ‘gauche’ conformation) of component i, the total energy of a binary mixture is given by

(16) $$E=-rN\tilde{\rho }\varepsilon^{*}+N_{1}\left(r_{1}-2\right)f_{1}\Delta \varepsilon_{g1}+N_{2}\left(r_{2}-2\right)f_{2}\Delta \varepsilon_{g2}$$

and the flex factor is given by

(17) $$f_{i}=\frac{\left(z-2\right)\exp\left(-\Delta \varepsilon_{g1}/RT\right)}{1+\left(z-2\right)\exp\left(-\Delta \varepsilon_{g1}/RT\right)}$$

The system entropy is given by

(18) $$S=-rNR\left\{\begin{array}{l} \left(\tilde{v}-1\right)\ln\left(1-\tilde{\rho }\right)+\frac{\ln\tilde{\rho }}{r}+\frac{\phi_{1}}{r_{1}}\ln\frac{\phi_{1}}{r_{1}}+\frac{\phi_{2}}{r_{2}}\ln\frac{\phi_{2}}{r_{2}}+\\ 1+\frac{\ln\left(2/Z\right)-1}{r}+\frac{\phi_{1}}{r_{1}}\left(r_{1}-2\right)\left[\ln\left(1-f_{1}\right)-f_{1}\frac{\Delta \varepsilon_{g1}}{RT}\right]+\\ +\frac{\phi_{2}}{r_{2}}\left(r_{2}-2\right)\left[\ln\left(1-f_{2}\right)-f_{2}\frac{\Delta \varepsilon_{g2}}{RT}\right] \end{array}\right\}$$

The only unknown parameter in this equation is the flex energy, Δ*ε_gi_*. In practice, ordinary solvents are considered fully flexible molecules and their Δ*ε_gi_* is set equal to zero. In the LF framework, the Δ*ε_gi_* of glassy polymers is determined based on the concept that the glassy state is, essentially, a frozen liquid or amorphous state and thus adopting the *Gibbs-Di Marzio criterion*, which states that the system entropy approaches zero as the system temperature approaches the glass transition temperature, T_g_, during cooling [29]. Thus, the flex energy for the polymer is obtained by setting *S* = 0 at T = T_g_. 

Otherwise, the phase equilibrium calculations in polymer-fluid systems at high temperatures through the glass transition temperature are carried out by setting the chemical potential of the fluid in the pure fluid state (Equation (13)) equal to that of the fluid in the mixture (Equation (12)).

### 2.3. Glassy Polymer–Compressed Fluid Systems

One point that has attracted considerable interest is the description of the phase equilibrium of polymer–solvent systems down to the glass transition region and over a broad range of external pressures and temperatures, especially when the solvent is a supercritical fluid, such as carbon dioxide. As mentioned above, these phase equilibrium calculations are of significant technological interest for the production of nano-foamed, micro-foamed, macro-foamed, or cellular polymeric materials [9,10,11,12,13,14]. The associated phase diagrams, however, may be quite peculiar.

**Figure 3:**
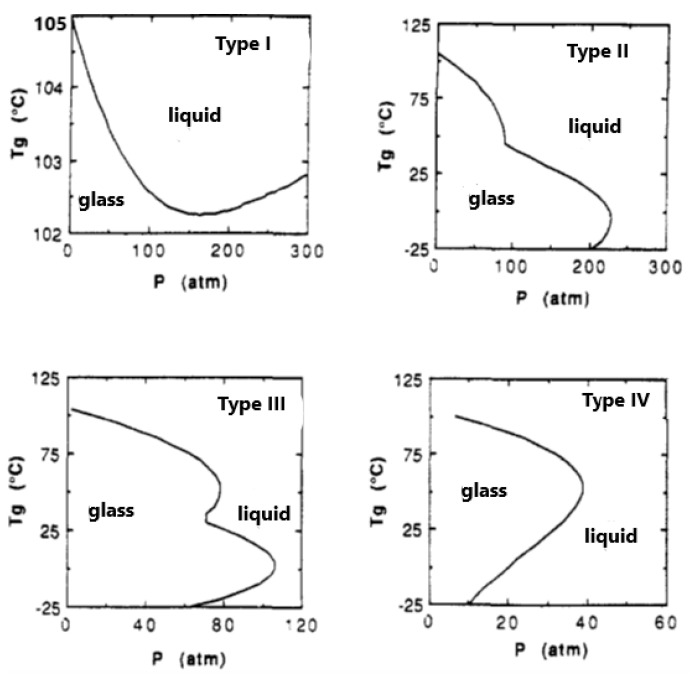
The four types of T_g_ behavior as a function of pressure, as predicted by the LF model. Reprinted with permission from ref. [13].

In order to understand the peculiarity of the phase diagrams of glassy polymer mixtures, we should first recall the effect of pressure on Tg. As shown in Figure 1, an increase in pressure reduces the free volume and restricts the free motion of the chain, resulting in a quasi-frozen state and thus increasing the value of T_g_. On the other hand, the sorbed fluid in the polymer matrix will act as a plasticizer and will tend to reduce Tg. Most often, a linear change in Tg with increasing solvent concentration in the mixture is observed [13]. However, in the case of compressed or supercritical fluids, the polymer matrix is subject, simultaneously, to high pressure and to plasticization by the dissolved fluid. The Tg of the mixture or the phase diagram, in this case, results from the interplay of these two opposing factors. Example diagrams, shown in Figure 3, illustrate the CO_2_-PMMA system. These diagrams are general and are not exclusive to the CO_2_-PMMA system.

As shown in Figure 3, there are four distinct types of *T_g_*–vs.–*P* diagrams, with the appropriate choice depending on the solvent solubility in the polymer matrix. The scaling constants for solvent and polymer in this figure are those of CO_2_ and PMMA (*T_g_* = 105 °C) and the solubility varies with the value of the ζ_12_ parameter.

The three types of diagrams with pressure maxima presented in Figure 3 are particularly interesting. Let us consider, as an example, the diagram of type IV. As observed, at an external pressure of 20 atm and at 120 °C, the system is in the liquid state. Upon cooling to a temperature of 75 °C, the system enters the glassy state and remains in that state down to ca. 0 °C. Upon further cooling, the system reenters the liquid state. This peculiar phenomenon is called *retrograde vitrification*. This phenomenon is also seen in Type II and Type III systems.

The system types shown in the diagrams of Types II, III and IV are very commonly used in the production of foams. Nucleation and foaming take place in the liquid state and cell stabilization takes place in the glassy state, for example, via rapid depressurization and removal (vaporization) of the supercritical solvent. As mentioned above, sorption in the glassy state is not a thermodynamic-equilibrium phenomenon and its description requires some further modification of the LF model. This description will be presented in a later section. First, however, the extension of the LF model needed to account for strong specific intermolecular interactions, such as hydrogen bonding (HB), will be examined in the next section.

## 3. Glass Transition and Hydrogen—Bonding: The LFHB and NRHB Models

The LF model has been extended to an equation-of-state model that accounts for hydrogen-bonding or Lewis acid–base interactions via a generalization of the Veytsman statistics [26,30,31] and is known in the literature as the LFHB (lattice fluid with hydrogen bonding) model. The most fundamental concepts will be reviewed here.

Let us consider a mixture in which there are m different kinds of hydrogen-bond (HB) donors and n kinds of HB acceptors. Let $d_{i}^{k}$ be the number of HB donors of type i (i = 1, m) in each molecule of type k (k = 1, 2) and $\alpha_{j}^{k}$ the number of HB acceptors of type j (j = 1, n) in each molecule of type k. The total number $N_{d}^{i}$ of HB donors i in the system is given by

(19) $$N_{d}^{i}=\sum_{k}^{2}d_{i}^{k}N_{k}$$

and the total number $N_{a}^{j}$ of HB acceptors j by

(20) $$N_{a}^{j}=\sum_{k}^{2}\alpha_{j}^{k}N_{k}$$

In addition,

(21) $$N_{i0}=N_{d}^{i}-\sum_{j}^{n}N_{ij}$$

(22) $$N_{0j}=N_{a}^{j}-\sum_{i}^{m}N_{ij}$$

where *Ν_i_*_0_ is the number of free (non-hydrogen-bonded) donor sites of type I and *Ν*_0*j*_ the number of free acceptor groups of type j, and *N*_ij_ is the number of hydrogen bonds between HB donors of type i and HB acceptors of type j.

The total HB energy *Ε_HB_* of the system is given by

(23) $$E_{HB}=\sum_{i}^{m}\sum_{j}^{n}N_{ij}E_{ij}^{H}$$

and the total number of hydrogen bonds is

(24) $$N_{H}=\sum_{i}^{m}\sum_{j}^{n}N_{ij}$$

where $E_{ij}^{H}$ is the corresponding HB energy of the i-j interaction contact.

The HB contribution, *G_HB_*, to the system Gibbs energy, is given by

(25) $$\frac{G_{HB}}{RT}=rN\left\{\sum_{i}^{m}\sum_{j}^{n}\nu_{ij}\left[1+\frac{G_{ij}^{H}}{RT}+\ln\left(\frac{\tilde{\upsilon }\nu_{ij}}{\nu_{i0}\nu_{0j}}\right)\right]+\sum_{i}^{m}\nu_{d}^{i}\ln\frac{\nu_{i0}}{\nu_{d}^{i}}+\sum_{i}^{m}\nu_{a}^{j}\ln\frac{\nu_{0j}}{\nu_{a}^{j}}\right\}$$

where

(26) $$\nu_{ij}\equiv \frac{N_{ij}}{rN}\nu_{i0}\equiv \frac{N_{i0}}{rN}\nu_{d}^{i}\equiv \frac{N_{d}^{i}}{rN}$$

etc.

The *v_ij_* values are obtained by minimization of Gibbs free energy, which yields m x n quasi-chemical-reaction equilibrium conditions:

(27) $$G_{ij}^{H}=-RT\ln\left(\frac{\tilde{\upsilon }\nu_{ij}}{\nu_{i0}\nu_{0j}}\right)$$

Which, in turn, simplifies the HB equation for Gibbs free energy to the following form:

$$\frac{G_{HB}}{RT}=rN\left\{\sum_{i}^{m}\sum_{j}^{n}\nu_{ij}+\sum_{i}^{m}\nu_{d}^{i}\ln\frac{\nu_{i0}}{\nu_{d}^{i}}+\sum_{i}^{m}\nu_{a}^{j}\ln\frac{\nu_{0j}}{\nu_{a}^{j}}\right\}=rN\left\{\nu_{H}+\sum_{i}^{m}\nu_{d}^{i}\ln\frac{\nu_{i0}}{\nu_{d}^{i}}+\sum_{i}^{m}\nu_{a}^{j}\ln\frac{\nu_{0j}}{\nu_{a}^{j}}\right\}$$

Derivation of Equation (25) with N_1_ will give the contribution of HB to the chemical potential of component **1**, which is as follows:

(28) $$\frac{\mu_{1,HB}}{RT}=r_{1}\nu_{H}-\sum_{i}^{m}d_{i}^{1}\ln\frac{\nu_{d}^{i}}{\nu_{i0}}-\sum_{j}^{n}a_{j}^{1}\ln\frac{\nu_{a}^{j}}{\nu_{0j}}$$

Equation (25) has very little effect on the equation of state, Equation (11), except on the 1/r term, which is replaced by

(29) $$\frac{1}{\bar{r}}=\frac{1}{r}-\sum_{i}^{m}\sum_{j}^{n}\nu_{ij}=\frac{1}{r}-\nu_{H}$$

where *ν_H_* is the reduced total number of hydrogen bonds in the system, found as $\nu_{H}=N_{H}/rN$.

The previous sections presented the role of the entropy equation in describing the glass transition and the thermodynamics of the glassy state. The usefulness of this equation is further enhanced by the inclusion of the HB contribution in associated polymer systems; the equations for all basic thermodynamic quantities include two main components: the HB contribution, which arises from hydrogen-bonding interactions, and the *physical* contribution, which represents all other types of intermolecular interactions, such as the LF equations described in the previous sections.

As with the other thermodynamic quantities, the entropy equation consists of two parts, the physical contribution, *S_P_*, and the HB contribution, *S_H_*. Equation (18) is used to calculate *S_P_*. The HB contribution is obtained from Equation (25) by derivation with temperature.

An interesting application of this equation is the calculation of the effect of HB on the glass transition behavior of polymer mixtures [20]. For a mixture of self-associated polymer 1 (*d*_1_ donor sites, *a*_1_ acceptor sites) with cross-associated polymer 2 (*a*_2_ acceptor sites), *S_H_* is given by

(30) $$\frac{S_{H}}{RrN}=-v_{H}+\frac{\phi_{1}d_{1}}{r_{1}}\ln\frac{\phi_{1}d_{1}}{\phi_{1}d_{1}-r_{1}v_{H}}+\frac{\phi_{1}a_{1}}{r_{1}}\ln\frac{\phi_{1}a_{1}}{\phi_{1}a_{1}-r_{1}v_{11}}+\frac{\phi_{2}a_{2}}{r_{2}}\ln\frac{\phi_{2}a_{2}}{\phi_{2}a_{2}-r_{2}v_{12}}\;$$

where *rν_H_* is the total number of hydrogen bonds per mol and *rν*_11_ and *rν*_12_ are the corresponding numbers of hydrogen bonds of type donor 1–acceptor 1 and donor 1–acceptor 2, respectively.

**Figure 4:**
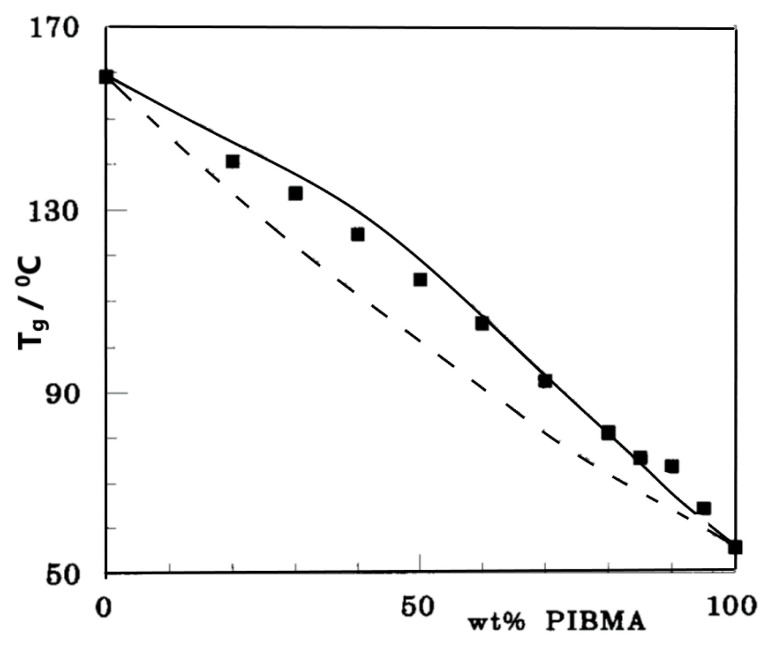
Glass transition temperatures of a SVPh60/PIBMA mixture. Filled rectangles represent experimental data [20]. The equation of the solid line was calculated from the LFHB model. The dashed line represents the non-hydrogen bonding LF contribution to T_g_. Reproduced with permission from reference [20].

The experimental T_g_s of the mixture of copolymer styrene-co-vinyl-phenol (SVPh60) and poly(iso-butyl methacrylate) (PIBMA) and the calculated T_g_s with and without the HB contribution are compared in Figure 4. As seen in this figure, the effect of HB is significant in this mixture. In fact, the HB contribution explains the unusual positive deviation of mixture T_g_s from a linear relationship with composition [20].

### NRHB and Retrograde Vitrification

One advanced version of the LFHB model accounts also for the non-random distribution of molecular species and free volume in the system and is known in the literature as the NRHB (non-randomness and hydrogen bonding) model [18,32,33]. The essentials of the NRHB model are given in the Supplementary Information (SI).

Let zq be the number of external contacts per molecule and *l* a measure of the non-linearity of the molecule given by

(31) $$l=\frac{z}{2}\left(r-q\right)+1-r$$

The NRHB equation of state is given by

(32) $$EOS=\frac{\tilde{P}\tilde{v}}{\tilde{T}}+\tilde{\rho }\ln\left(1-\tilde{\rho }\right)+z\tilde{\rho }\ln\Gamma_{0}-{\tilde{\rho }}^{2}\left(\frac{l}{r}-v_{H}\right)-\frac{z}{2}\tilde{\rho }\ln\left[1-\tilde{\rho }+\frac{q}{r}\tilde{\rho }\right]=0$$

and the NRHB equation for the chemical potential is given by

(33) $$\begin{array}{l} \frac{\mu_{k}}{RT}=\ln\frac{\varphi_{k}}{\delta_{k}r_{k}}-r_{k}\sum_{j}\frac{\varphi_{j}l_{j}}{r_{j}}+\ln\tilde{\rho }+r_{k}\left(\tilde{v}-1\right)\ln\left(1-\tilde{\rho }\right)-\\ -\frac{z}{2}r_{k}\left[\tilde{v}-1+\frac{q_{k}}{r_{k}}\right]\ln\left[1-\tilde{\rho }+\frac{q}{r}\tilde{\rho }\right]+\frac{zq_{k}}{2}\left[\ln\Gamma_{kk}+\frac{r_{k}}{q_{k}}\left(\tilde{v}-1\right)\ln\Gamma_{00}\right]-\frac{q_{k}}{{\tilde{T}}_{k}}+r_{k}\frac{\tilde{P}\tilde{v}}{\tilde{T}}\frac{v_{k}^{*}}{v^{*}}+\\ +\frac{\mu_{k,HB}}{RT}+DR\left(EOS\right) \end{array}$$

where *μ_k_*_,*HB*_ is given by Equation (28) and *DR*, as in Equation (12), is given by

(34) $$DR=-rN\tilde{v}\left(\frac{\partial \tilde{\rho }}{\partial N_{1}}\right)$$

Once again, the last term on the right-hand side of Equation (33) is zero only at equilibrium conditions.

This NRHB model, along with the above Gibbs-DiMarzio criterion of zero entropy at the glass transition, have been used by Scherilo et al. [34] to successfully describe the retrograde vitrification (type IV diagram in Figure 3) of a polystyrene-toluene system using carefully conducted experimental measurements [35]. This work indicates that the diagrams shown in Figure 3 show common behaviors, rather than behaviors exclusive to glassy polymers with compressed fluids. In fact, the maximum pressure in the type IV diagram was about 40 mbar [34,35].

The above analysis is useful for estimating the glass transition temperature as a function of pressure or composition. When the system is in the glassy state, the calculations must account for the non-equilibrium character of the system. This aspect is addressed by the generalized non-equilibrium lattice-fluid (NELF) model, which is discussed in the next section.

## 4. Sorption, Solubility, Dilation in Glassy Polymer Systems and the NELF Model

As mentioned in Section 1, the thermodynamics of the glassy polymer state raises a series of challenging issues. The temperature and pressure alone are not sufficient to determine the state of the glassy polymer. As the state changes with time due to relaxation or aging, the sorption or solubility of a given solute at a fixed temperature and pressure may also vary with time. This variability is particularly challenging when the mixture remains in the glassy state while the sorbed penetrant causes changes in the range of relaxation times of the polymer. Nonetheless, some progress has been made in modeling these changes with reasonable approximations.

One of the most successful approaches to modeling the thermodynamics of the glassy state was that by Doghieri and Sarti [21]. Their central assumption was that the thermodynamic state of the glassy polymer system is determined once its density is known or considered known. In this way, the polymer density is treated as the “order parameter” of the non-equilibrium system. Their assumption was incorporated into the lattice-fluid model, generating the successful NELF model. Their approach was further extended subsequently [3,17,22,34,35] and is now widely used in the literature as the NET-GP (non-equilibrium thermodynamics of glassy polymers) model. As this is the main approach to equation-of-state in the field, we will focus on it in the remainder of this section. However, for coherence with the above discussion, the definitions and the version of NELF by Boudouris and Panayiotou [36] will also be discussed. The inclusion of these ideas will facilitate the rationalization of the ad hoc assumptions of NET-GP.

### 4.1. Definitions of Key Quantities in NELF Model

Let us consider a fluid-penetrant (1)–polymer (2) system and, following Doghieri and Sarti [21], let us introduce the polymer density, *ρ*_2_, as the order parameter in the penetrant/glassy polymer system. Experimentally, a weighted polymer sample of mass *m*_2_ is placed in the sorption cell and the amount of sorbed penetrant, *m_1_*, is measured. Often, the volume of the swollen sample, *V*_s_, is measured, which may give the *apparent* density of the polymer, *ρ*_2_ *= m*_2_*/V_s_*. The word apparent is underlined because it is tacitly assumed that the measured swollen volume is identical to the volume of the polymer under the conditions of the measurement. The density, *ρ*, of the mixture is then given by the following equation:

(35) $$\rho =\frac{m_{2}+m_{1}}{V_{0}+\Delta V}=\frac{m_{2}+m_{1}}{V_{s}}=\frac{m_{2}}{V_{s}}\frac{m_{2}+m_{1}}{m_{2}}=\frac{\rho_{2}}{w_{2}}$$

The experimental data are typically reported as isotherms of the sorbed penetrant per g of polymer, *s*_1_ *= m*_1_*/m*_2_, as a function of the (partial) pressure, *P*_1_, in the cell at equilibrium. For clarity of discussion, we will confine ourselves to the case of a pure-component penetrant fluid. Extension to multicomponent penetrants is complex but rather straightforward. In our case, the following equation describes the system:

(36) $$s_{1}=\frac{m_{1}}{m_{2}}=\frac{w_{1}}{w_{2}}=\frac{w_{1}}{1-w_{1}}$$

Thus, the weight fraction of the penetrant, *w*_1_, is given in terms of the measured s_1_ by

(37) $$w_{1}=\frac{s_{1}}{1+s_{1}}$$

When the volume of the sample is also measured, the reported experimental data are typically the relative volume changes, Δ*V*/*V*_0_, with respect to the initial volume, *V*_0_, of the polymer sample. In terms of the above quantities, this volume change may be expressed as follows:

(38) $$\frac{\Delta V}{V_{0}}=\frac{\left(1+s_{1}\right)\rho_{2}^{0}}{\rho }-1$$

where $\rho_{2}^{0}$ is the pure polymer density. Equation (38) may be rewritten as follows:

(39) $$\frac{\Delta V}{V_{0}}=\frac{w_{2}\left(1+s_{1}\right)\rho_{2}^{0}}{\rho_{2}}-1=\frac{\rho_{2}^{0}}{\rho_{2}}-1$$

The simplicity of Equation (39) explains why the quantity, *ρ*_2_, although somewhat ill-defined, is attractive to use in modelling sorption in glassy polymers. In fact, as observed, the information content of *ρ*_2_ is equivalent to that of the volume change Δ*V*/*V*_0_. Thus, for a polymer sample of known (pure polymer) density, if *ρ_2_* is considered to be known, Δ*V*/*V*_0_ is also known, and vice versa, or

(40) $$\rho_{2}=\rho_{2}^{0}/\left(1+\frac{\Delta V}{V_{0}}\right)$$

An alternative approach, which would bypass the definition of the apparent *ρ*_2_, is the direct physical definition of the system density *ρ* [36]:

(41) $$\rho =\frac{m_{2}+m_{1}}{V_{0}+\Delta V}=\frac{m_{2}}{V_{0}}\frac{1+s_{1}}{1+\Delta V/V_{0}}=\rho_{2}^{0}\frac{1+s_{1}}{1+m_{1}V_{12}/V_{0}}=\rho_{2}^{0}\frac{1+s_{1}}{1+s_{v1}V_{12}}=\frac{\rho_{2}^{0}}{w_{2}+w_{1}V_{12}\rho_{2}^{0}}$$

where *V*_12_ is the proportionality coefficient between the volume change, Δ*V*/*V*_0_, and the degree of sorption per unit volume, *s_v_*_1_ *= m*_1_/*V*_0_. We will see soon the advantage of this alternative approach and of using Equation (41). What is important to notice is that to use the Doghieri/Sarti approach [21], we must know the initial density of the pure glassy polymer, as well as the (apparent) density of the polymer at any degree of sorption. In the alternative approach [36], the information needed is the density of the pure glassy polymer and the proportionality constant V_12_ for a limited range of pressure changes. This approach leaves room for limited predictive calculations. 

Equation (41) is, in essence, equivalent to defining the volume dilation as follows:

(42) $$\frac{\Delta V}{V_{0}}=V_{12}\frac{m_{1}}{V_{0}}=V_{12}s_{v1}$$

This equation is useful when the experimental data are reported as the amount of sorbed gas under STP conditions per cubic centimeter of initial polymer sample. In the usual case, when this initial volume is 1 cm^3^, *s_v_*_1_ *= m*_1_ in g of penetrant per cm^3^ of polymer. Dividing *m*_1_ by the molar mass *M*_1_ of the penetrant and multiplying by 22,433, *s_v_*_1_ gives the amount of sorbed gas under STP conditions per cubic centimeter of initial polymer sample.

Equation (42), implies that, for a constant *V*_12_, when the amount of sorption is known, the volume change is also known, and vice versa. Again, *V*_12_ is, in general, constant over a limited range of external conditions.

The two expressions of the extent of sorption, *s*_1_ and *s_v_*_1_, are related as follows:

(43) $$\frac{s_{v1}}{s_{1}}=\frac{m_{1}}{V_{0}}\frac{m_{2}}{m_{1}}=\frac{m_{2}}{V_{0}}=\rho_{2}^{0}$$

Thus, Equation (42) may be rewritten alternatively as

$$\frac{\Delta V}{V_{0}}=V_{12}\rho_{2}^{0}s_{1}$$

The extent of sorption is, however, dictated by the pressure at equilibrium. Thus, it would be useful to re-express this Equation in terms of pressure. This may be accomplished by employing Henry’s law at low concentrations: *H = y*_1_*P*/*c*_1_, which gives the concentration of a pure-component penetrant (y_1_ = 1):

(44) $$c_{1}=\frac{P}{H}$$

with *c*_1_ *= m*_1_/*V*. If the density of the pure glassy polymer is $\rho_{2}^{0}$, the initial volume is $V^{0}=m_{2}/\rho_{2}^{0}$.

Then,

(45) $$\frac{\Delta V}{V}=\frac{\Delta V}{V^{0}}\frac{V^{0}}{V}=s_{v1}V_{12}\frac{V^{0}}{V}=\left(\frac{m_{1}}{V^{0}}\right)\frac{V^{0}}{V}V_{12}=\frac{m_{1}}{V}V_{12}=c_{1}V_{12}$$

Substituting variables from Equation (44), we obtain:

(46) $$\frac{\Delta V}{V}=P\left(\frac{V_{12}}{H}\right)=K_{12}P$$

Thus, by holding K_12_ constant, Equation (46) gives the relative volume change at any external pressure. This is important information; in order to appreciate its importance, let us divide both sides of Equations (39) and (46) to obtain:

$$\frac{\Delta V/V^{0}}{\Delta V/V}=\frac{V}{V^{0}}=\frac{\rho_{2}^{0}}{\rho_{2}}=\left[\frac{\rho_{2}^{0}}{\rho_{2}}-1\right]/K_{12}P$$

Rearranging, we obtain

$$K_{12}P\frac{\rho_{2}^{0}}{\rho_{2}}=\frac{\rho_{2}^{0}}{\rho_{2}}-1\;or\;\frac{\rho_{2}^{0}}{\rho_{2}}=\frac{1}{1-K_{12}P}$$

or

(47) $$\rho_{2}=\rho_{2}^{0}\left(1-K_{12}P\right)$$

Later, Baschetti et al. [22] proposed the following empirical relation for the change in density, ρ_2_, of the glassy polymer as a function of pressure:

(48) $$\rho_{2}=\rho_{2}^{0}\left(1-k_{s}P\right)$$

which is Equation (47) with K_12_ replaced by the *swelling constant*, *k_s_*., or, by setting:

(49) $$k_{s}=\frac{V_{12}}{H}$$

This equation interconnects the constants of the two NELF approaches [21,36] and rationalizes the ad hoc Equation (48) via Henry’s law (constant, H). As observed, although *k_s_* is named the *swelling* constant, it is in essence a combined constant that integrates volume change (*V*_12_) with sorption or solubility (*H*). We will come back to this combined constant below.

Substituting variables from Equation (48) in Equation (40), we obtain

(50) $$\frac{\Delta V}{V_{0}}=\frac{k_{s}P}{1-k_{s}P}$$

or

(51) $$k_{s}P=\frac{\Delta V}{V_{0}}/\left(1+\frac{\Delta V}{V_{0}}\right)=\frac{\Delta V}{V}$$

Thus, if *k_s_* is considered a known constant independent of pressure, Equation (50) directly gives the volume change as a function of pressure. With these definitions, we may now proceed to sorption calculations using the NELF model.

### 4.2. NELF Model Calculations

In this subsection, we will review sorption and swelling calculations in glassy polymer systems via the NELF approach, which amounts to introducing system density as an *order* parameter or *internal state* parameter to the above lattice-fluid model [21,36]. This parameter will let us account for the extent to which the system state departs from equilibrium. We will start with the calculations reported in ref. [36].

The central underlying idea of the NELF approach is the non-zero value of the EOS terms in Equation (12), or the invalidity of Equation (11) under non-equilibrium conditions. One must introduce the information on density, *ρ*, from external sources. Replacing this value in Equation (11) will give the value of the EOS term to be substituted in Equation (12). The extent of sorption is then obtained by setting the chemical potential of the penetrant, component 1, in the pure-fluid state (Equation (13)) equal to that of the penetrant in the mixture (Equation (12)). The required scaling constants for penetrant and polymer are, typically, obtained from the literature or by correlating pure-component (equation-of-state) experimental data.

The system studied in ref. [36] is carbon dioxide-polycarbonate (PC), for which there is extensive experimental information available on both sorption and swelling of unconditioned and conditioned polymer samples [37,38]; the required data for PC and CO_2_ are available in refs. [39,40,41,42], respectively.

**Figure 5:**
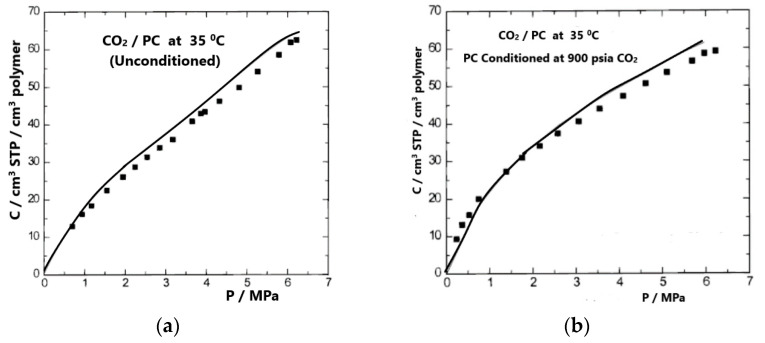
Experimental [37] (symbols) and calculated (lines) gas solubilities of CO_2_ in unconditioned (**a**) and conditioned (**b**) PC at 35 °C, obtained by considering the excess volume to be known. Reproduced, with permission, from reference [36].

**Figure 6:**
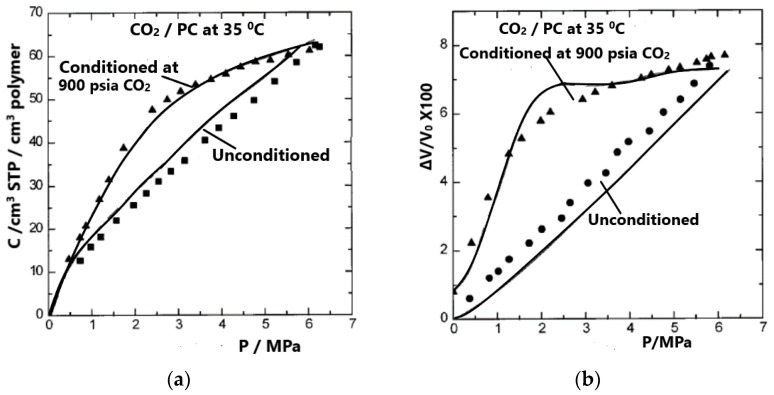
Experimental [37] (symbols) and calculated (lines) gas solubilities on unconditioned and conditioned PC samples, considering volume changes to be known (**a**) and volume changes in the same sample calculated by considering gas solubilities to be known (**b**). Reproduced with permission from [36].

Two types of calculations were carried out in ref. [36]: one using as an intermediate quantity the proportionality constant, *V*_12_, and the other using Equation (42) and setting the chemical potential of the penetrant in the pure state equal to that of the penetrant in the mixture. This set of two equations includes three unknowns, *ρ*, *V*_12_, and composition (weight fraction, *w*_1_). The value of *V*_12_ and the composition was determined, in the first type of calculation, by considering the density or the volume change (swelling), which was known (from experimental data). In the second type of calculation, the sorption was considered known (from the experiment) and the volume change was obtained by solving the above set of two equations. These calculations do not require any adjustable parameters, and the calculations are predictions of the NELF model. The results thus obtained are shown in Figure 5 and Figure 6. The density of the unconditioned sample was set equal to 1.200 g/cm^3^ [37]. The conditioned sample, with an initial volume of 1 cm^3^, had a volume of 1.008 cm^3^ after removal of the sorbed CO_2_. Thus, the density of the conditioned sample was set equal to 1.200/1.008 = 1.190 g/cm^3^.

Figure 5 compares the experimental solubilities of CO_2_ in the “as-received” glassy PC sample and in the conditioned PC sample at 35 °C. The calculations were carried out by considering the swelling (volume dilation), Δ*V*/*V*^0^*,* which was known [37]. A similar calculation to obtain volume dilations was carried out by considering the gas solubilities to be known.

One calculation of particular interest involves the simultaneous calculation of sorption on an unconditioned sample and desorption from a conditioned PC sample. The result of such a calculation is shown in Figure 6, which compares experimental [37] and calculated gas solubilities (considering volume change to be known) and volume changes (considering sorption values or solubilities to be known). In view of the non-equilibrium character of the system, the comparison shown in Figure 6 reveals a highly satisfactory result.

**Figure 7:**
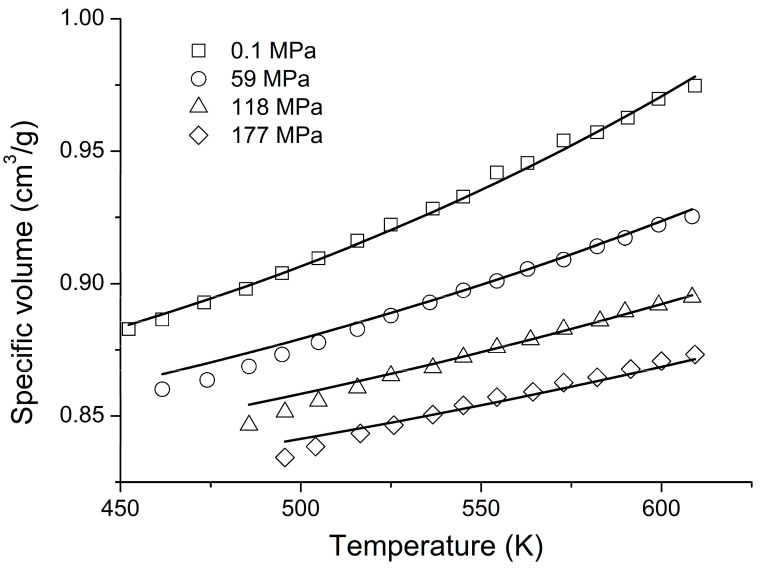
NRHB model fitting of PVT data [40] to obtain the scaling constants for PC.

An interesting alternative type of calculation is typically carried out within the framework of the NET-GP model [3,21,22] by adjusting the binary interaction parameter ξ_12_ (cf. Equation (9)). This type of calculation is probably the one most widely used with NET-GP at present. Here, calculations using both the LFHB and NRHB models will be presented for comparison. The scaling constants are reported in Table 1. The NRHB scaling constants for PC were obtained by fitting experimental PVT data [40], as shown in Figure 7.

**Figure 8:**
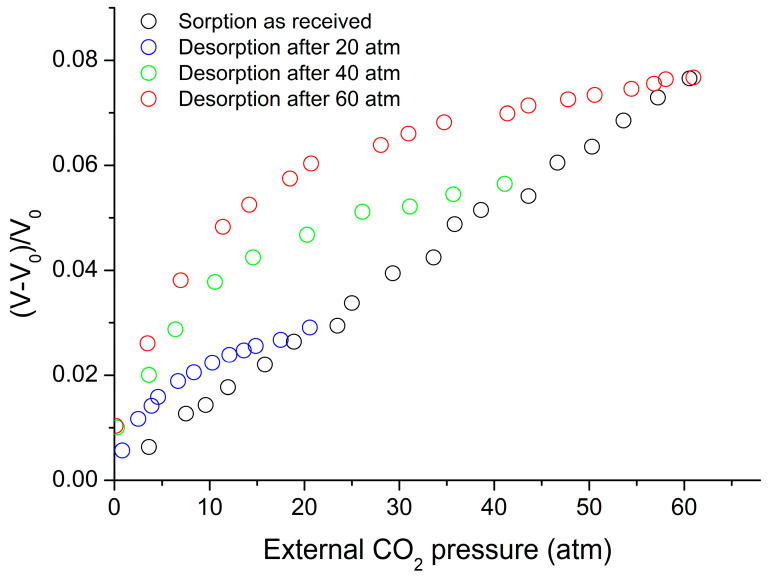
Changes in PC-CO_2_ volume during sorption and desorption at 35 °C, ρ_2_ = 1.200 g/cm^3^ or V_0_ = 0.8333 cm^3^/g [38].

**Figure 9:**
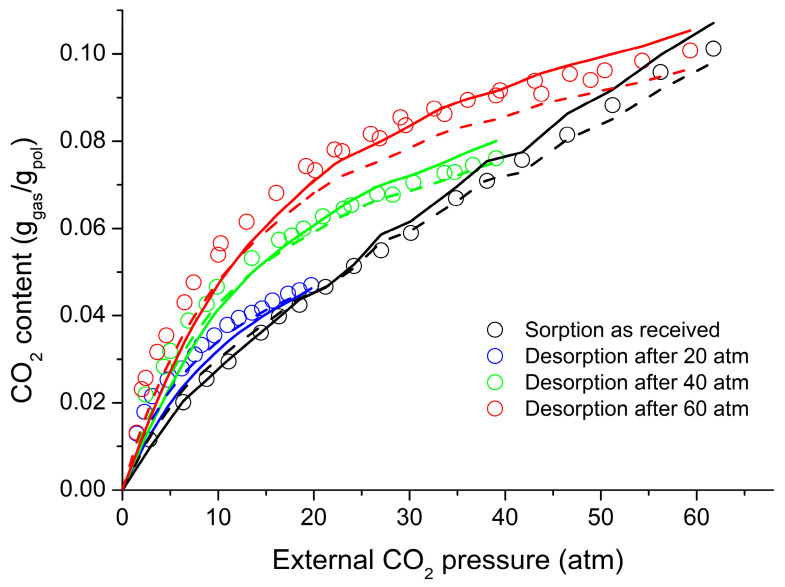
Comparison of CO_2_ sorption/desorption data [38] in PC at 35 °C with NE-LFHB (solid lines) (correlation of only sorption data with ξ = 1.1533) and NETGP-NRHB (dashed lines) (correlation of only sorption data with ξ = 1.1444). In both models, the values of the mixture volume are taken from Figure 8.

Figure 8 shows the experimental data for volume changes upon sorption and desorption of CO_2_ in the system with PC at 35 °C [38]. These data were considered to be known and were used in the sorption calculations reported in Figure 9. The sorption data given in Figure 9 were correlated and the binary parameter ξ_12_ was adjusted for the best fit, while the desorption data were predicted based on the values of ξ_12_; The predicted values are included in the same figure. The predictions of the non-equilibrium LFHB and NRHB models are nearly equally satisfactory. The fitted parameters are ξ_12_ = 1.1533 for LFHB and ξ_12_ = 1.1444 for NRHB. Both values depart significantly from unity. We will discuss these departures below.

**Figure 10:**
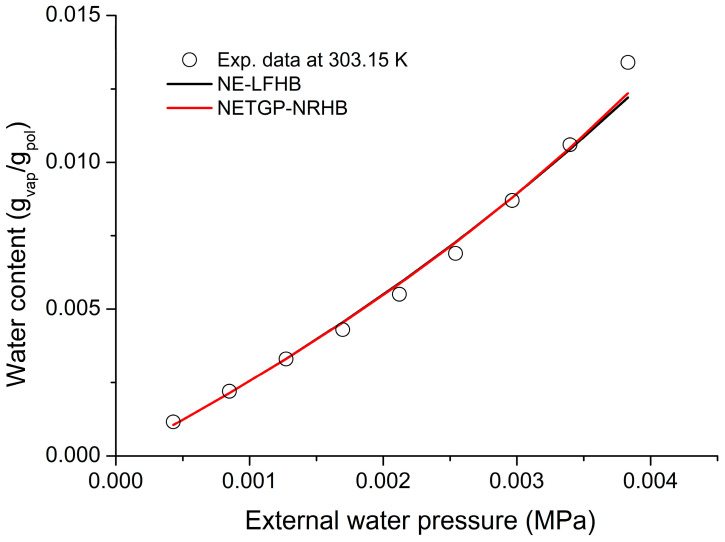
Comparison of H_2_O sorption data [43] in 6FDA_6FpDA at 30 °C by NE-LFHB (correlation of sorption data with ξ = 0.8211 and G_12_ = −12,773 J mol^−1^) and NETGP-NRHB (correlation of sorption data with ξ = 0.869 and G_12_ = −12,100 J mol^−1^).

The parameters reported in Table 2 and Table 3 are taken from ref. [43]. The density of the glassy polymer is 1.5781 g cm^−3^ [43]. For both models, two adjustable parameters were used: the interaction parameter, ξ, and the Gibbs energy of formation of the cross-6FDA_6FpDA-H_2_O hydrogen bonding, G_12_. These values are reported in the caption of Figure 10.

Similar work was carried out with the non-equilibrium (NET-GP) NRHB model to correlate predictions with data on water sorption by other glassy polymers used in engineering and to predict the extent of the various hydrogen-bonding interactions involved. These approaches yielded good results and are reviewed in ref. [18]. Of course, the NET-GP approach may be used with other equation-of-state models. Calculations similar to those above, as an example, have been carried out using the PC-SAFT model [19].

**Figure 11:**
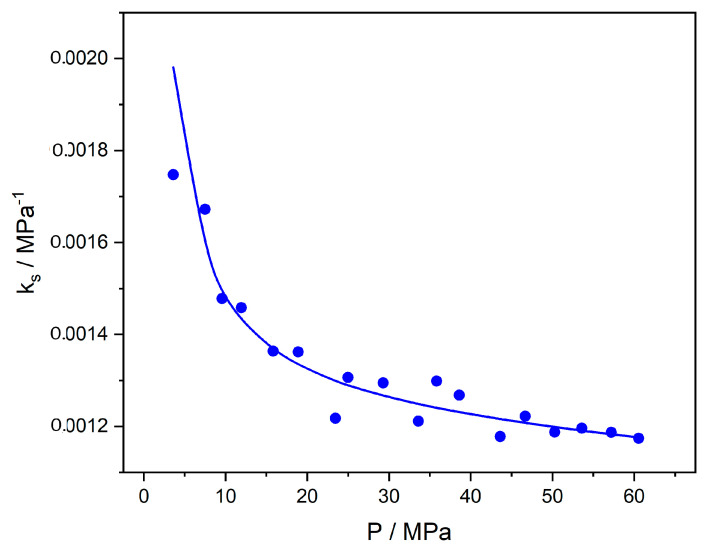
The swelling constant as a function of pressure in the CO_2_-PC system, as obtained from Equation (51) and the experimental ΔV/V_0_ data [37] at 35 °C. The line is obtained from the correlated ΔV/V_0_ data.

Another alternative type of NET-GP calculations adopts Equation (48) to acquire the apparent density of the glassy polymer as a function of pressure and run calculations as above, usually by adjusting ξ_12_ [3,17,22]. However, one should keep in mind that the swelling constant, *ks*, is not really a constant. To understand the degree of variation in this value, we may use the above data for the CO_2_-PC system [37] and calculate *k_s_* directly from the (experimentally known) ΔV/V_0_ data by using Equation (51). The results are shown in Figure 11. As observed, *k_s_* is not a constant and varies significantly with pressure. Sarti et al. [3,44,45] and others [17,18,34,35,43] take an average value for *k_s_* in order to correlate sorption data, typically by adjusting the cross-interaction parameter, *ξ*_12_.

As discussed above, in these non-equilibrium LFHB and NRHB thermodynamic calculations, the *ξ*_12_ values typically depart rather significantly from unity, and this departure leaves little room for obtaining insights into molecular thermodynamics. Such results also indicate that the scaling constants being used may not be appropriate. Importantly, the scaling constants of the glassy polymer are invariably those obtained from the correlation of PVT data at temperatures well above glass transition. This method of obtaining constants raises doubts regarding their appropriateness for describing sorption/swelling in glassy polymers. The fact that the selected order parameter in the above NET-GP approach is the polymer density may also indicate that the scaling constants from the liquid/rubber state are inappropriate for use in density calculations in the glass state.

DeAngelis and Sarti [44,45] have applied the NET-GP approach to the solubility of gas and liquid solutes in glassy polymers by using Equation (48) to estimate the density of the glassy polymer. The solubilities were then correlated by adjusting their binary interaction (which is analogous to) ξ_12_, which was found to be much closer to unity, at around 1.03. This value might be a much more acceptable binary parameter from which to obtain insights into molecular dynamics. Sarti et al. use a variation on the LF formalism; their binary interaction parameter corrects the cross-pressure difference Δ*P*_12_*, rather than the cross-interaction energy *ε*_12_*. The results may be influenced, however, by the simultaneous use of the “swelling” constant *k_s_* of Equation (48). As mentioned above, this constant combines features of volume change with the degree of sorption/solubility. The authors carried out careful calculations and verified that even at the highest values of solubility, the system remained in the glassy state. Thus, on the basis of their very good correlation results, they seem to be justified in claiming that the scaling constants obtained from the liquid state may apply in the glass state as well. Thus, the combination of NET-GP and Equation (48) appears to be reasonably well justified. There are, however, some considerations that must be borne in mind when analyzing these calculations.

The NELF approach assigns the same scaling constants to the glassy polymer and to the rubbery polymer. This choice, however, may not be fully justified, especially for the scaling density, *ρ*_2_*, as discussed in the Supplementary Information (SI). The equation of state (Equation (11)) should not be used with data for the non-equilibrium glassy polymer, but it may be used to give a qualitative picture of the scaling density that is compatible with the experimental PVT data for the glassy state. Such calculations are reported in the Supplementary Information (SI). As has been shown, the use of *ρ*_2_* values from the rubbery material for the glassy polymer may not be justified at low sorption values. Due to this misuse of the *ρ*_2_* scaling constant, it is reasonable to expect ξ_12_ values that depart from unity. However, more systems must be studied before we can safely draw conclusions on this issue.

## 5. Conclusions

A molecular equation-of-state model, like the generalized lattice-fluid model discussed in this work, may be used for thermodynamic calculations in the glassy polymer state, as well as for estimations of the variation in glass transition temperature with changes in external conditions, such as mixture composition or pressure. Peculiar relevant phenomena, like retrograde vitrification, may be sufficiently modeled with the LF model. The role of strong specific intermolecular interactions, such as hydrogen bonding, may also be evaluated by this model using either the LFHB or NRHB version. In addition to retrograde vitrification, another peculiarity, the positive departure from linearity of the glass transition temperatures of (miscible) polymer mixtures, is also well predicted by the LFHB model. NET-GP seems to be versatile for modeling sorption and dilation in the glassy polymer state via non-equilibrium thermodynamics. The use of the empirical Equation (48) [22] and its swelling constant, *k_s_*, are rationalized on the basis of Henry’s law.

## Figures and Tables

**Table 1:** LFHB and NRHB lattice-fluid parameters.

		LFHB			NRHB		
	T * (K)	P * (MPa)	*ρ* * (g cm^−3^)	ε_h_ * (J mol^−1^)	ε_s_ * (J mol^−1^ K^−1^)	v_sp,0_ * (cm^3^ g^−1^)	s
PC	755	534	1.275	7973.4	−2.8371	0.77968	0.728
CO_2_	300	630	1.515	3468.4	−4.5855	0.79641	0.909

**Table 2:** Lattice-fluid parameters from the LFHB and NRHB models.

		LFHB			NRHB		
	T * (K)	P * (MPa)	*ρ* * (g cm^−3^)	ε_h_ * (J mol^−1^)	ε_s_ * (J mol^−1^ K^−1^)	v_sp,0_ * (cm^3^ g^−1^)	s
6FDA_6FpDA	750.1	476.5	1.806	5471.1	3.8652	0.5174	0.7757
H_2_O	484.1	452.7	1.0647	5336.5	−6.506	0.9703	0.8610

**Table 3:** Parameters of water-water hydrogen bonding from the LFHB and NRHB models.

		LFHB			NRHB	
	E_11_ (J mol^−1^)		S_11_ (J mol^−1^ K^−1^)	E_11_ (J mol^−1^)		S_11_ (J mol^−1^ K^−1^)
H_2_O	−18,424		−19.83	−16,100		−14.7

## Data Availability

Data are available within this article and Supplementary Material.

## Supplementary Materials

The following supporting information can be downloaded at: https://www.mdpi.com/article/10.3390/polym16020298/s1, Figure S1: Experimental and calculated specific volumes of the GlassVF of PVAc by the LF equation of state using the scaling constants reported in Table S1; Figure S2: The variation of ρ2* with penetrant pressure obtained by reproducing experi-mental sorption data from corresponding data on volume changes in the system CO_2_-PC at 35 °C; Table S1: LFHB scaling constants for PVAc from experimental PVT data; Table S2: LFHB scaling constants for PC from experimental PVT data. Refs. [46,47] have been cited in Supplementary Material.

## References

[1] Gee G. (1970). The glassy state in polymers. Contemp. Phys..

[2] Hutchinson J.M., Haward R.N., Young R.J. (1997). Relaxation processes and physical aging. The Physics of Glassy Polymers.

[3] Minelli M., Sarti G.C. (2020). 110th Anniversary: Gas and Vapor Sorption in Glassy Polymeric Membranes—Critical Review of Different Physical and Mathematical Models. Ind. Eng. Chem. Res..

[4] Roth C., Matyjaszewski K., Gnanou Y., Hadjichristidis N., Muthukumar M. (2022). Polymer glasses. Macromolecular Engineering: From Precise Synthesis to Macroscopic Materials and Applications.

[5] de Gennes P.G. (1979). Scaling Concepts in Polymer Physics.

[6] McKinney J.E., Goldstein M. (1974). PVT relationships for liquid and glassy poly(vinyl acetate). J. Res. Nat. Bur. Std..

[7] McKinney J.E., Simha R. (1977). Thermodynamics of the densification process for polymer glasses. J. Res. Nat. Bur. Std..

[8] Dixon D., Luna-Bárcenas G., Johnston K.P. (1994). Microcellular microspheres and microballoons by precipitation with a vapour-liquid compressed fluid antisolvent. Polymer.

[9] Tomasko D.L., Burley A., Feng L., Yeh S.K., Miyazono K., Nirmal-Kumar S., Kusaka I., Koelling K. (2009). Development of CO_2_ for polymer foam applications. J. Supercrit. Fluids.

[10] Siripurapu S., DeSimone J.M., Khan S.A., Spontak R.J. (2004). Low temperature Surface Mediated Foaming of Polymer Films. Adv. Mater..

[11] Zhou Y., Tian Y., Peng X. (2023). Applications and Challenges of Supercritical Foaming Technology. Polymers.

[12] Wissinger R.G., Paulaitis E. (1991). Glass transitions in polymer/CO_2_ mixtures at elevated pressures. J. Polym. Sci. Part B Polym. Phys..

[13] Condo P.D., Sanchez I.C., Panayiotou C.G., Johnston K.P. (1992). Glass transition behavior including retrograde vitrification of polymers with compressed fluid diluents. Macromolecules.

[14] McHugh M.A., Krukonis V.J. (1986). Supercritical Fluid Extraction: Principles and Practice.

[15] Pope D.S., Koros W.J. (1992). Effect of Various Preexposure Agents on Methane Sorption and Dilation in Tetramethyl Polycarbonate. Macromolecules.

[16] Parsons A., Heater K., Randal P. Evaluation of Supercritical CO_2_ Spray Technology as a Cost Effective Approach to Reduction of Solvents in Wood Finishing. Proceedings of the AIChE Conference.

[17] Galizia M., Stevens K.A., Smith Z.P., Paul D.R., Freeman B.D. (2016). Non-equilibrium lattice fluid modeling of gas solubility in HAB-6FDA polyimide and its thermally rearranged analogs. Macromolecules.

[18] Mensitieri G., Scherillo G., Panayiotou C., Musto P. (2020). Towards a predictive thermodynamic description of sorption processes in polymers: The synergy between theoretical EoS models and vibrational spectroscopy. Mater. Sci. Eng. R.

[19] Borrmann D., Danzer A., Sadowski G. (2022). Water Sorption in Glassy Polyvinylpyrrolidone-Based Polymers. Membranes.

[20] Prinos J., Panayiotou C. (1995). Glass-transition temperatures in hydrogen-bonded polymer mixtures. Polymer.

[21] Doghieri F., Sarti G.C. (1996). Nonequilibrium lattice fluids:  A predictive model for the solubility in glassy polymers. Macromolecules.

[22] Baschetti G.M., Doghieri F., Sarti G.C. (2001). Solubility in glassy polymers: Correlations through the non-equilibrium lattice fluid model. Ind. Eng. Chem. Res..

[23] Sanchez I.C., Lacombe R.H. (1976). An elementary molecular theory of classical fluids. Pure fluids. J. Phys. Chem..

[24] Sanchez I.C., Lacombe R.H. (1978). Statistical thermodynamics of polymer solutions. Macromolecules.

[25] Rodgers P.A., Sanchez I.C. (1993). Improvement to the Lattice-Fluid Prediction of Gas Solubilities in Polymer Liquids. J. Polymer Sci. Part B Polymer Phys..

[26] Sanchez I.C., Panayiotou C., Sandler S. (1994). Equations of state thermodynamics of polymer and related solutions. Models for Thermodynamic and Phase Equilibria Calculations.

[27] Flory P.J. (1942). Thermodynamics of high polymer solutions. J. Chem. Phys..

[28] Flory P. (1973). Polymer Chemistry.

[29] Gibbs J.H., Di Marzio E.A. (1958). Nature of the glass transition and the glassy state. J. Chem. Phys..

[30] Panayiotou C., Sanchez I.C. (1991). Hydrogen bonding in fluids: An equation of-state approach. J. Phys. Chem..

[31] Veytsman B.A. (1990). Are Lattice Models Valid for Fluids with Hydrogen Bonds?. J. Phys. Chem..

[32] Panayiotou C., Tsivintzelis I., Economou I.G. (2007). Nonrandom Hydrogen-Bonding Model of Fluids and their Mixtures. 2. Multicomponent Mixtures. Ind. Eng. Chem. Res..

[33] Panayiotou C., Acree W.E., Zuburtikudis I. (2023). COSMO-RS and LSER models of solution thermodynamics: Towards a COSMO-LSER equation of state model of fluids. J. Mol. Liq..

[34] Scherillo G., Loianno V., Pierleoni D., Esposito R., Brasiello A., Minelli M., Doghieri F., Mensitieri G. (2018). Modeling Retrograde Vitrification in the Polystyrene−Toluene System. J. Phys. Chem. B.

[35] Pierleoni D., Minelli M., Scherillo G., Mensitieri G., Loianno V., Bonavolontà F., Doghieri F. (2017). Analysis of polystyrene-toluene system through ‘dynamic’ sorption tests: Glass transitions and retrograde vitrification. J. Phys. Chem. B.

[36] Boudouris D., Panayiotou C. (1998). On the solubility of gas molecules in glassy polymers and the non-equilibrium approach. Macromolecules.

[37] Fleming G.K., Koros W.J. (1986). Dilation of polymers by sorption of carbon dioxide at elevated pressures. 1. Silicone rubber and unconditioned polycarbonate. Macromolecules.

[38] Fleming G.K., Koros W.J. (1990). Carbon dioxide conditioning effects on sorption and volume dilation behavior for bisphenol A-polycarbonate. Macromolecules.

[39] Olabisi O., Simha R. (1975). Pressure-volume-temperature studies of amorphous and crystallizable polymers. I. Experimental. Macromolecules.

[40] Zoller P. (1982). A Study of the Pressure-Volume-Temperature Relationships of Four Related Amorphous Polymers: Polycarbonate, Polyarylate, Phenoxy, and Polysulfone. J. Polym. Sci. Polym. Phys. Ed..

[41] Zoller P., Walsh D.J. (1995). Standard Pressure Volume Temperature Data for Polymers.

[42] Vargaftik N.B. (1975). Handbook of Physical Properties of Liquids and Gases.

[43] Scherillo G., Sanguigno L., Galizia M., Lavorgna M., Musto P., Mensitieri G. (2012). Non-equilibrium compressible lattice theories accounting for hydrogen bonding interactions: Modelling water sorption thermodynamics in fluorinated polyimides. Fluid Phase Equilibr..

[44] De Angelis M.G., Sarti G.C. (2011). Solubility of Gases and Liquids in Glassy Polymers. Ann. Rev. Chem. Biomol. Eng..

[45] Sarti G.C., De Angelis M.G. (2012). Calculation of the Solubility of Liquid Solutes in Glassy Polymers. AIChE J..

[46] Panayiotou C., Vera J.H. (1982). Thermodynamics or r-mer Fluids and their Mixtures. Polymer J..

[47] Panayiotou C., Stefanis E., Tsivintzelis I., Pantoula M., Economou I. (2004). Nonrandom Hydrogen-Bonding Model of Fluids and Their Mixtures 1. Pure Fluids. Ind. Eng. Chem. Res..

