# Supplementary material for: Thermodynamics of the Glassy Polymer State: Equilibrium and Non-Equilibrium Aspects
Source: Polymers (Basel). 2024 Jan 22;16(2):298. doi: 10.3390/polym16020298 (PMC10820664; doi:10.3390/polym16020298)
Supplement: Supplementary file 1 [file polymers-16-00298-s001.zip › polymers-2801798-supplementary.pdf]

polymers

MDPI

Supplementary Information

Thermodynamics of the Glassy-Polymer State: Equilibrium and non-Equilibrium Aspects

Costas Panayiotou

Department of Chemical Engineering, Aristotle University of Thessaloniki, 54624 Thessaloniki, Greece

Part I: The scaling constants of glassy polymers

There are plenty of PVT data for glassy polymers in the open literature [1 - 5] and it is tempting to use the equilibrium equation of state (equation 11 of the main text) to correlate as well the PVT data for the glassy polymers. Since the molecular constitution and structure of the polymer chain does not change in going from the rubbery to the glassy state, the scaling constants for the interaction energy, $\varepsilon_0*+(T-298.15)\varepsilon_1*$ [6], are not expected to change either. The PVT data for the glassy polymer may, then, be correlated by permitting a change to ρ* only. Although this is thermodynamically unacceptable, it may still be useful for an appreciation of the departure from equilibrium of the glassy state. The question is, then, how the formation history of the glassy polymer may be taken into account in a value of ρ*. Before proceeding, we must answer this question.

In their seminal work, McKinney and Goldstein [1] have followed three alternative formation routes for the glassy poly(vinyl acetate) (PVAc) and report carefully conducted PVT measurements for the glassy states as well as for the rubbery state. This set of data is well suited for answering the above question. For this purpose, the PVT data for the rubbery state were, first, correlated in order to obtain the scaling constants in the typical way of NRHB model [7 - 9] or the PV model [6]. These scaling constants are reported in Table S1. Subsequently, the PVT data of the glassy states were correlated by keeping the scaling constants ε* and ε_s* identical to those of the rubber state. Only the scaling constant for the density ρ* or the specific volume, $v_{sp}* = 1/\rho*$, was changing in the glassy state. The scaling constants for the three types of glassy PVAc are also reported in Table S1. *GlassVF* was formed by changing the pressure in the liquid region and then cooling isobarically the sample at a constant rate of 5 ^{0}C/h. *Glass1atm* was formed by isobaric cooling at 1 atm down to the desired temperature in the glassy state followed by a pressure increase (consecutive pressure jumps) up to the desired pressure. *Glass800* was formed by isobaric cooling at 800 bar down to the desired temperature followed by depressurization down to the desired pressure.

Table S1. LFHB scaling constants for PVAc from experimental PVT data [1]. The scaling constants vary with temperature and pressure in the NRHB manner: $\varepsilon* = \varepsilon_0* + (T-298.15)\varepsilon_1*$ and $v_{sp}* = v_{sp0}* + 0.00015(T-298.15)A - 0.000135BP$.

	ε_0*	ε_1*	v_{sp0}*	*A*	*B*	v_{sp}* *at 260 K*
**PVAc State**						
Liquid / Rubber	5269	3.050	0.7913	1.000	1.000	*0.7856*
GlassVF	5269	3.050	0.7934	-0.508	0.723	0.7963
Glass1atm	5269	3.050	0.7935	-0.530	0.263	0.7965
Glass800	5269	3.050	0.7881	-0.509	0.217	0.7911

In the last column of Table S1 are reported the specific hard-core volumes, v_{sp}*, at 260 K for the three glasses as well as for a fictitious (subcooled) liquid of scaling constants the ones corresponding to the liquid / rubber state of PVAc. As observed, even the glass

formed at the highest pressure has a specific volume v_{sp}* higher than the corresponding volume of the (fictitious) liquid. It is these higher specific hard-core volumes of the glassy state, which enable the equation-of-state model to describe properly the actual densities or specific volumes of the glassy state. An example of these calculations is shown in Figure S1 for the GlassVF.

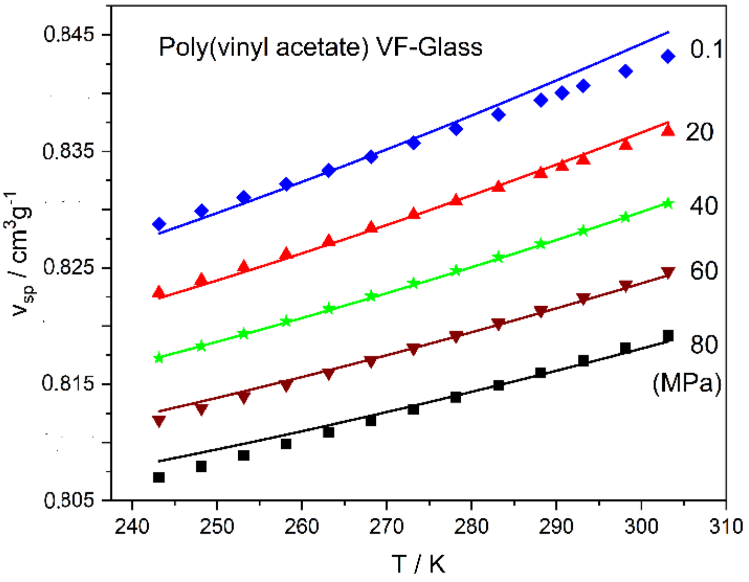

Figure S1. Experimental [1] (symbols) and calculated (lines) specific volumes of the GlassVF of PVAc by the LF equation of state using the scaling constants reported in Table S1.

Experimental PVT data for the glassy PC are also available [3 - 5] and the above calculations may also be done for PC. This was done and the resulting scaling constants are reported in Table S2. In the last column of this table are shown the values of v_{sp}* at 35 ^{0}C. As seen, the hard-core specific volume of the glassy PC is again rather significantly higher than the corresponding volume for the (fictitious) liquid.

Table S2. LFHB scaling constants for PC from experimental PVT data [5]. The scaling constants vary with temperature and pressure as in Table S1.

	ε_0*	ε_1*	v_{sp0}*	*A*	*B*	v_{sp}* *at 308 K*
**PC State**						
Liquid / Rubber	5829	2.390	0.7838	1.000	1.000	*0.7853*
Glass	5829	2.390	0.7983	-0.674	0.099	0.7973

The properties of the glassy polymer state depend on its formation history, and it was not expected to obtain universal values for A and B in Tables S1 and S2 as is obtained for the rubbery polymers. Nevertheless, there is a qualitatively common behavior regarding v_{sp}* and, as shown, the glassy polymers exhibit rather significantly higher than the corresponding volume for the (fictitious) liquid/rubber at the same temperature well below Tg. This may explain the large departures from unity of the binary parameter ξ_{12}, discussed in the main text, when using values of v_{sp}* of the fictitious liquid for the glassy polymer. The problem is that this scaling constant depends on the formation history of the glass as shown in Table S1. The PC sample, as an example, of Fleming and Koros [10],

especially, the conditioned ones, would require even higher values for $v_{sp}*$ to reproduce their densities.

Nevertheless, at least qualitatively, we may also see how $\varrho^* = 1/v_{sp}^*$ varies with sorption extent or sorption pressure. With CO_2 scaling constants in the PV / NRHB practice (universal segment volume v* = 9.75 cm^3/mol, ε_0^* = 3166, ε_1^* = -2.234 and ϱ^*= 1.2812), we may use the above experimental sorption and swelling data [10] and obtain the value of ϱ_2^* which reproduces the sorption extent from volume changes and the inverse. Figure S2 is an example of such calculations. As seen, the use of the value ϱ_2^*= 1.2734 g/cm^3 from the rubbery / liquid state is a reasonable approximation for relatively high sorption pressures.

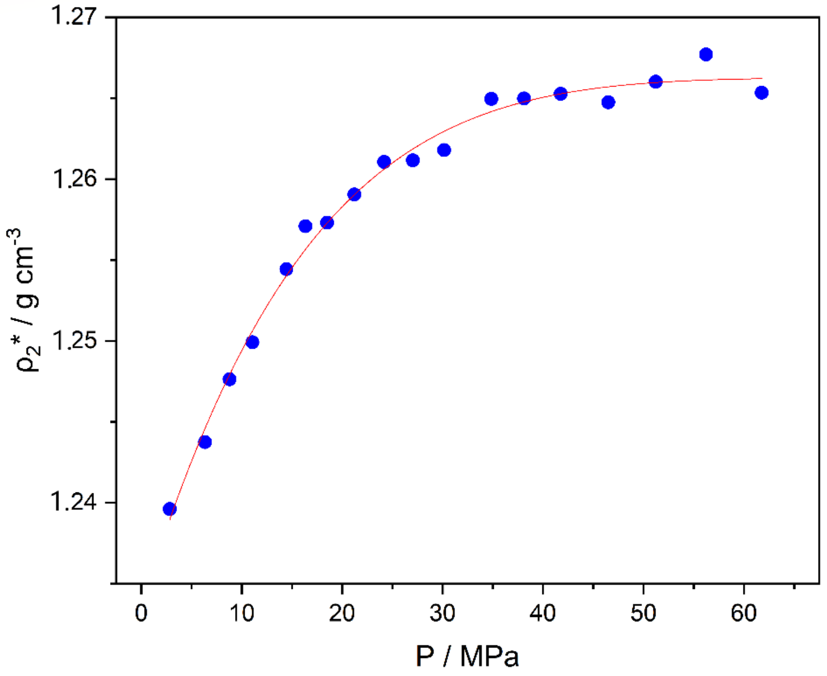

Figure S2. The variation of ϱ_2^* with penetrant pressure obtained by reproducing experimental [10] sorption data from corresponding data on volume changes in the system CO_2 - PC at 35 ^{0}C.

Part II: The essentials of the NRHB Model

As mentioned in the main text, one advanced version of the LFHB model which, besides hydrogen-bonding, accounts also for the non-random distribution of molecular species and free volume in the system is NRHB (Non-randomness and Hydrogen Bonding) model [7 - 9]. The NRHB equation for Gibbs free energy is given by (symbols as in the main text):

$$\frac{G}{RT} = rN\left\{ \begin{array}{l} -\frac{l}{r} - \frac{\tilde{\rho}}{\tilde{T}} + \frac{\tilde{P}\tilde{v}}{\tilde{T}} + (\tilde{v}-1)\ln(1-\tilde{\rho}) + \frac{\ln\tilde{\rho}}{r} + \\ \sum_{k=1}^{t}\frac{\phi_k}{r_k}\ln\frac{\phi_k}{\omega_k} - \frac{z}{2}\left(\tilde{v}-1+\frac{q}{r}\right)\ln\left(1-\tilde{\rho}+\frac{q}{r}\tilde{\rho}\right) \end{array} \right\}$$
$$+N\left\{\sum_{k=0}^{t}\frac{zq}{2}\theta_k\ln\left[\Gamma_{kk}\exp\left(\frac{-\varepsilon_{kk}}{RT}\right)\right]\right\} + \frac{G_{HB}}{RT} \quad \text{(S1)}$$

where, G_{HB} is again given by equation 25 of the main text. The non-randomness factors, Γ_{ij}, are defined as the ratio of the number of binary interactions, N_{ij}, in the real system over the corresponding number, N_{ij}^0, in a fictitious system of perfect randomness, or

$$\Gamma_{ij} = \frac{N_{ij}}{N_{ij}^0} = \Gamma_i \Gamma_j \exp\left(\frac{\varepsilon_{ij}}{RT}\right) \tag{S2}$$

For convenience the binary factor, Γ_{ij}, is often split in the segment-specific non-randomness factors, Γ_i and Γ_j [11], while ε_{ij} is the interaction energy for the contact i-j. These non-randomness factors are obtained by minimization of the Gibbs free energy with respect to the number of contacts Nij leading to Guggenheim's Quasi-chemical conditions [7]:

$$\frac{\Gamma_{kl}^2}{\Gamma_{kk}\Gamma_{ll}} = \exp\left(\frac{2\varepsilon_{kl} - \varepsilon_{kk} - \varepsilon_{ll}}{RT}\right) = \exp\left(\frac{\Delta\varepsilon_{kl}}{RT}\right) \tag{S3}$$

which is, in essence, the source of definition-equation S2 [11]. zq in equation S1 is the number of external contacts per molecule, l is a measure of the non-linearity of the molecule and is given by:

$$l = \frac{z}{2}\left(r - q\right) + 1 - r \tag{S4}$$

and the surface fraction, θ_k, in the actual system is defined by:

$$\theta_k = \frac{N_k q_k}{qN}\frac{qN}{N_q} = \Theta_k \frac{qN}{N_q} = \Theta_k \frac{\sum_j q_j N_j}{N_0 + \sum_j q_j N_j} = \Theta_k \frac{q/r}{q/r + \tilde{v} - 1} \tag{S5}$$

where, Θ_k, is the corresponding surface fraction in a system without empty sites ($N_0 = 0$).

The NRHB equation of state is given by:

$$EOS = \frac{\tilde{P}\tilde{v}}{\tilde{T}} + \tilde{\rho}\ln\left(1 - \tilde{\rho}\right) + z\tilde{\rho}\ln\Gamma_0 - \tilde{\rho}^2\left(\frac{l}{r} - v_H\right) - \frac{z}{2}\tilde{\rho}\ln\left[1 - \tilde{\rho} + \frac{q}{r}\tilde{\rho}\right] = 0 \tag{S6}$$

and the NRHB equation for the chemical potential is given by:

$$\begin{aligned} \frac{\mu_k}{RT} = {} & \ln\frac{\varphi_k}{\delta_k r_k} - r_k \sum_j \frac{\varphi_j l_j}{r_j} + \ln\tilde{\rho} + r_k\left(\tilde{v} - 1\right)\ln\left(1 - \tilde{\rho}\right) - \\ & - \frac{z}{2} r_k \left[\tilde{v} - 1 + \frac{q_k}{r_k}\right]\ln\left[1 - \tilde{\rho} + \frac{q}{r}\tilde{\rho}\right] + \frac{zq_k}{2}\left[\ln\Gamma_{kk} + \frac{r_k}{q_k}\left(\tilde{v} - 1\right)\ln\Gamma_{00}\right] - \frac{q_k}{\tilde{T}_k} + r_k \frac{\tilde{P}\tilde{v}}{\tilde{T}}\frac{v_k^*}{v^*} + \\ & + \frac{\mu_{k,HB}}{RT} + DR\left(EOS\right) \end{aligned} \tag{S7}$$

where, $\mu_{k,HB}$ is given by equation 28 of the main text and DR, as in equation 12 of the main text, is given by:

$$DR = -rN\tilde{v}\left(\frac{\partial\tilde{\rho}}{\partial N_1}\right) \tag{S8}$$

The last term on the right-hand side of equation S7 is zero at equilibrium only conditions.

References

1. McKinney, J.E., and Goldstein, M. 1974. PVT relationships for liquid and glassy poly(vinyl acetate). *J. Res. Nat. Bur. Std*. 78A: 331 – 353.
2. McKinney, J.E., and Simha, R. 1977. Thermodynamics of the densification process for polymer glasses. *J. Res. Nat. Bur. Std*. 81A: 283 – 297.
3. Olabisi, O., and Simha, R. 1975. Pressure-volume-temperature studies of amorphous and crystallizable polymers. I. Experimental. *Macromolecules* 8: 206 – 210
4. Zoller, P. A 1982. Study of the Pressure-Volume-Temperature Relationships of Four Related Amorphous Polymers: Polycarbonate, Polyarylate, Phenoxy, and Polysulfone. *J. Polym. Sci., Polym. Phys. Ed*., 20, 1453.
5. Zoller,P., Walsh,D.J. 1995. *Standard Pressure Volume Temperature Data for Polymers.* Technomic Publishing AG. Basel.
6. Panayiotou, C., Vera, J.H. 1982, Thermodynamics or r-mer Fluids and their Mixtures, *Polymer J*., 14, 681- 694.
7. Panayiotou, C., Stefanis, E., Tsivintzelis, I., Pantoula, M., Economou, I. 2004. Nonrandom Hydrogen-Bonding Model of Fluids and Their Mixtures 1. Pure Fluids. *Ind. Eng. Chem. Res*. 43, 6592-6606
8. Panayiotou, C., Tsivintzelis, I., Economou., I.G. 2007. Nonrandom Hydrogen-Bonding Model of Fluids and their Mixtures. 2. Multicomponent Mixtures *Ind. Eng. Chem. Res*. 46, 2628 - 2636.
9. Mensitieri, G., Scherillo, G.. Panayiotou, C., Musto, P. 2020. Towards a predictive thermodynamic description of sorption processes in polymers: The synergy between theoretical EoS models and vibrational spectroscopy, *Materials Science & Engineering R*, 140, 100525
10. a) Fleming, G. K.; Koros, W. J. Dilation of polymers by sorption of carbon dioxide at elevated pressures. 1. Silicone rubber and unconditioned polycarbonate. Macromolecules 1986, 19, 2285; b) Fleming, G. K., and Koros, W. J. 1990. Carbon dioxide conditioning effects on sorption and volume dilation behavior for bisphenol A-polycarbonate *Macromolecules* 23: 1353-1360.
11. Panayiotou, C., Acree, W.E., Zuburtikudis, I., 2023. COSMO-RS and LSER models of solution thermodynamics: Towards a COSMO-LSER equation of state model of fluids, *J. Molec. Liquids* 390, 122992

Disclaimer/Publisher's Note: The statements, opinions and data contained in all publications are solely those of the individual author(s) and contributor(s) and not of MDPI and/or the editor(s). MDPI and/or the editor(s) disclaim responsibility for any injury to people or property resulting from any ideas, methods, instructions or products referred to in the content.
